# SIRPα-antibody fusion proteins stimulate phagocytosis and promote elimination of acute myeloid leukemia cells

**DOI:** 10.18632/oncotarget.14500

**Published:** 2017-01-04

**Authors:** Laia Pascual Ponce, Nadja C. Fenn, Nadine Moritz, Christina Krupka, Jan-Hendrik Kozik, Kirsten Lauber, Marion Subklewe, Karl-Peter Hopfner

**Affiliations:** ^1^ Gene Center Munich, Department of Biochemistry, Ludwig-Maximilians-Universität München, Munich, Germany; ^2^ Department of Internal Medicine III, Klinikum der Universität München, Ludwig-Maximilians-Universität München, Munich, Germany; ^3^ Gene Center and Clinical Co-operation Group Immunotherapy at the Helmholtz Zentrum München, Munich, Germany; ^4^ Department of Radiation Oncology, Klinikum der Universität München, Ludwig-Maximilians-Universität München, Munich, Germany; ^5^ Graduate School of Quantitative Biosciences Munich, Ludwig-Maximilians-Universität München, Munich, Germany

**Keywords:** therapeutic antibody, immunotherapy, CD47, SIRPα, acute myeloid leukemia

## Abstract

CD47, expressed on a variety of tumor cells, confers immune resistance by delivering an inhibitory “don't eat me” signal to phagocytic cells via its myeloid-specific receptor SIRPα. Recent studies have shown that blocking the CD47-SIRPα axis with CD47-directed antibodies or antibody-derivatives enhances phagocytosis and increases antitumor immune effects. However, CD47 expression on healthy cells creates an antigen sink and potential sites of toxicity, limiting the efficacy of CD47-directed therapies. In this study, we first characterized CD47 expression in Acute Myeloid Leukemia (AML) patients (*n* = 213) and found that CD47 is highly expressed on both AML bulk and stem cells irrespective of the disease state. Furthermore, to inhibit the CD47-SIRPα signaling pathway at the tumor site, we developed a so-called local inhibitory checkpoint *m*onoclonal antibody (licMAB) by grafting the endogenous SIRPα domain to the N-terminus of the light chain of an antibody targeting CD33, a surface antigen expressed in AML. LicMABs selectively bind CD33-expressing cells even in the presence of a large CD33-negative CD47-positive antigen sink, stimulate phagocytosis of AML cells and eliminate AML cell lines and primary, patient-derived AML cells. Our findings qualify licMABs as a promising therapeutic approach to confine the benefit of disrupting the CD47-SIRPα axis to tumor antigen-expressing cells.

## INTRODUCTION

The use of monoclonal antibodies (mAbs) has become an important and promising approach in cancer immunotherapy [1]. Their clinical success is based on their ability to specifically bind to antigens differentially expressed in cancer cells and to simultaneously recruit immune effector cells via interaction with their Fc receptors (FcRs). This triggers immune cell activation, including antibody-dependent cellular cytotoxicity (ADCC) and antibody-dependent cellular phagocytosis (ADCP) [2].

Recent studies suggest that a highly promising strategy for treating different types of cancers involves targeting not only the tumor cell but also immune checkpoints, which comprise a plethora of inhibitory pathways fundamental for the maintenance of self-tolerance under normal physiological conditions [3, 4]. It is well documented that tumor cells often utilize these immune checkpoints as a mechanism to escape immune recognition and gain immune resistance, demonstrating the necessity of target these molecular pathways [5].

CD47 is a transmembrane protein ubiquitously expressed on normal cells. Its receptor, the signal regulatory protein alpha (SIRPα), is expressed on phagocytic cells, including macrophages and dendritic cells (DCs). Upon binding to CD47, SIRPα transmits a “don't eat me” signal that inhibits phagocytosis, thus preventing normal host cells from being cleared by macrophages [6, 7]. However, it has been shown that the disruption of CD47-SIRPα interaction by a blocking antibody against human CD47 (B6H12.2) enables phagocytosis of Acute Myeloid Leukemia Stem Cells (AML LSC) and Acute Lymphoid Leukemia (ALL) cells *in vitro* and inhibits tumor engraftment *in vivo* [8, 9]. Moreover, B6H12.2 repressed tumor growth or cured solid tumors in mouse studies [10]. Additionally, a humanized IgG4 antiCD47 antibody (Hu5F9-G4) was pre-clinically evaluated and entered phase I trials (NCT02216409) in patients with AML and solid tumors in 2014 [11]. Hu5F9-G4 showed a potent enforcement of phagocytosis of primary human AML cells *in vitro* and an elimination of patient-derived AML xenografts *in vivo*. Furthermore, B6H12.2 and Hu5F9-G4 have been shown to synergize with tumor-specific monoclonal antibodies such as rituximab, an antiCD20 antibody, leading to inhibition of engraftment of Non-Hodgkin Lymphoma (NHL) and cure of xenografted mice [11, 12].

Nevertheless, the ubiquitous expression of CD47 on healthy cells, such as red blood cells (RBCs), which are abundant and accessible within the bloodstream, might create a large antigen sink and potential sites of toxicity based on unintended binding. The unwanted binding of CD47 on healthy cells could thus be minimized by reducing the binding strength for CD47 [13]. As the native interaction between SIRPα and CD47 is rather weak [14–16], the endogenous extracellular N-terminal domain of SIRPα, consisting of an immunoglobulin superfamily V-like fold [15, 16], is an attractive alternative to interfere with the CD47-SIRPα axis while avoiding the mentioned risks [17]. In agreement with this, TTI-621, a SIRPα-Fc fusion protein that uses the endogenous SIRPα domain to block the CD47-SIRPα axis, was developed by Trillium Therapeutics and is currently being evaluated in phase I trials for hematologic malignancies (NCT02890368).

CD33, a sialic acid-dependent cytoadhesion molecule, is a validated target in AML [18]. Its expression on AML cells and AML LSCs [18, 19] has made it the target antigen of choice for several antibody-based approaches, including mAbs, antibody-drug conjugates (ADC), bispecific T-cell engagers (BiTE), bispecific killer cell engagers (BiKE) and single-chain variable fragment triplebodies (sctbs) [20–27]. Albeit promising results were obtained in pre-clinical and clinical investigations of several therapeutic formats, no CD33-targeting agent have been yet accepted by none of the Medicine Regulatory Authorities. Thus, new CD33-directed strategies are needed in order to exploit its full potential.

Similarly to CD33, CD47 is overexpressed on AML bulk cells and AML LSCs [8, 28]. Expression of CD47 contributes to tumor progression by enabling AML cells to evade phagocytosis and is consequently correlated with a poor prognosis [8]. Hence, disruption of the CD47-SIRPα interaction might represent a therapeutic strategy in AML to induce clearance of tumor cells.

Here, we report the development and characterization of a new antibody format, hereafter called a local inhibitory checkpoint monoclonal antibody (licMAB). LicMABs were created by grafting the endogenous N-terminal Ig domain of SIRPα onto the variable light chain of a CD33-targeting IgG1 antibody scaffold, similar to the recently published “SIRPabodies” [17]. We suggest that the resulting licMAB blocks the CD47-SIRPα interaction at the site of tumor antigen-expressing cells. In other words, by targeting the tumor antigen CD33, the blockade of the “don't eat me” signal and therefore the antitumor activity, is restricted to CD33-expressing cells. Moreover, we propose that the blockade of CD47-SIRPα signaling can be enhanced by fusing a second SIRPα domain (resulting in a double SIRPα licMAB), thus increasing the local SIRPα concentration.

SIRPα-antiCD33 and 2xSIRPα-antiCD33 licMABs were evaluated based on their binding specificity, cytotoxic effect and phagocytosis of AML cells *in vitro*. LicMABs successfully stimulate phagocytosis of human AML cell lines and enforce elimination of human AML cell lines and primary, patient-derived AML cells. Thus, our studies establish licMABs as a possible therapeutic approach to locally deliver the benefit of CD47-SIRPα blockade to tumor cells.

## RESULTS

### CD47 is highly expressed on bulk AML cells and AML LSCs independent of disease state

**Figure 1:**
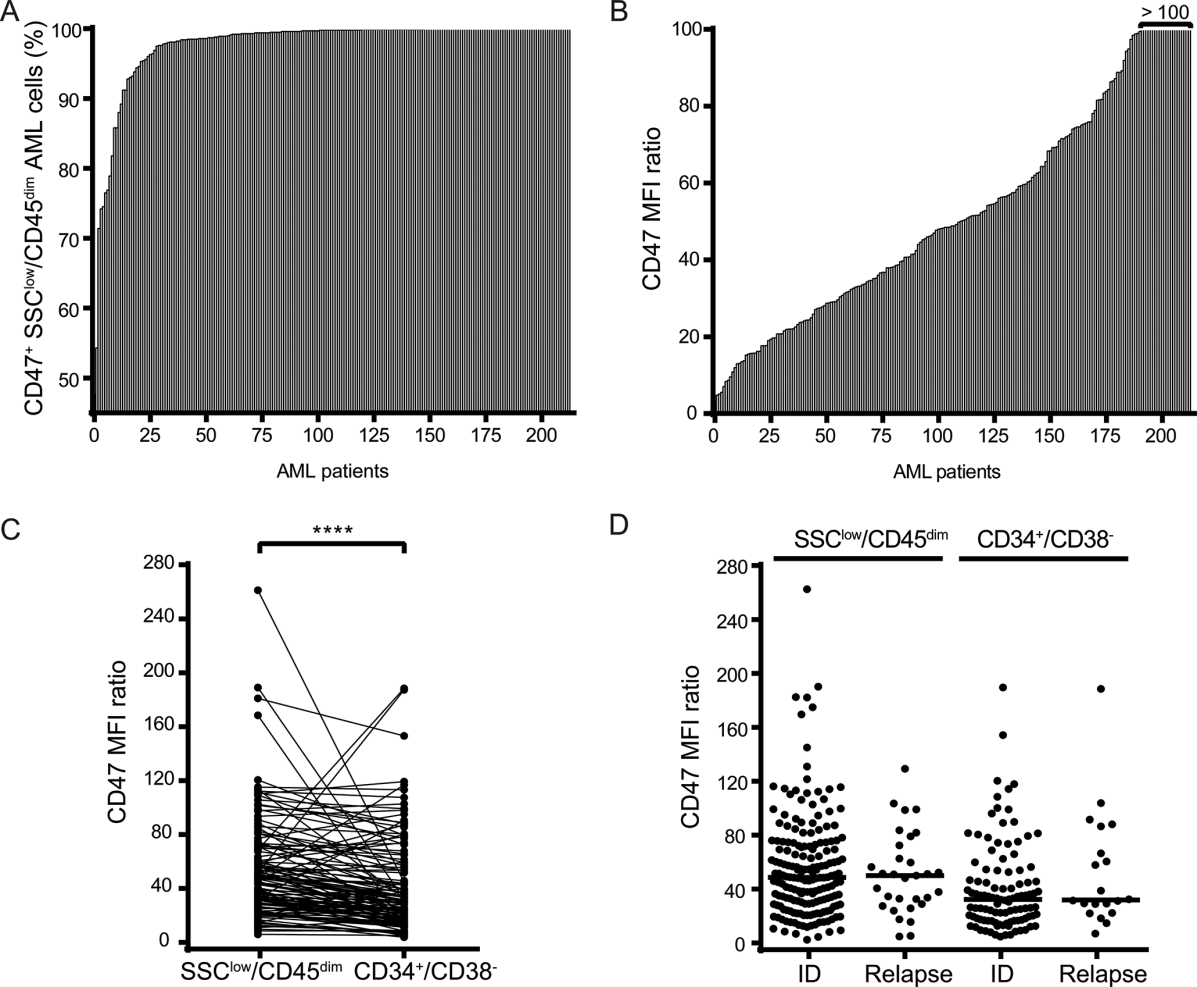
CD47 is highly expressed on bulk AML cells and AML LSCs irrespective of the disease state (**A**) Percentage of CD47 positive cells of SSC^low^/CD45^dim^ bulk AML cells from 213 AML patients. (**B**) CD47 expression levels (MFI ratio) on SSC^low^/SSC^low^/CD45^dim^ bulk AML cells (*n* = 213). (**C**) Comparison of CD47 MFI ratio between SSC^low^/CD45^dim^ bulk AML cells and CD34^+^/CD38^-^ AML LSCs (*n* = 213). (**D**) Comparison of CD47 MFI ratio at initial diagnosis (ID) and relapse in SSC^low^/CD45^dim^ bulk AML cells (*n* = 182 ID; = 31 relapse) and CD34^+^/CD38^-^ LSCs (*n* = 106 ID; = 20 relapse). Statistical differences were assessed by the Mann-Whitney *U* test (*p-value* < 0.0001****).

By expressing CD47, AML cells trigger the “don't eat me” signal to macrophages via SIRPα engagement and can therefore escape the immune system by inhibiting phagocytosis. Accordingly, previous studies have shown that CD47 is expressed at higher levels on AML LSCs than on their normal counterparts and it also correlates with poor clinical outcomes [8]. To further characterize CD47 expression on AML cells, we analyzed 213 AML patient samples; 182 of whom were newly diagnosed and 31 of whom in relapse (Figure 1).

CD47 expression could be detected on 99.8 % of bulk SSC^low^/CD45^dim^ AML cells from the 213 patient samples (Figure 1A). Despite considerable inter-patient heterogeneity, expression levels of CD47 were consistently high, resulting in a Median Fluorescence Intensity (MFI) ratio of 48.83 (Figure 1B).

After characterizing CD47 expression on bulk AML cells, we sought to evaluate the expression level of CD47 on AML LSCs, which are found within the CD34^+^/CD38^-^compartment of SSC^low^/CD45^dim^ cells and are believed to be the source of AML relapse. Although the expression level of CD47 was significantly lower on AML LSCs with respect to bulk AML cells, the expression level was high in both cell compartments (MFI ratios of 48.07 and 32.31, respectively) (Figure 1C). Furthermore, we compared the expression level of CD47 on bulk and AML LSCs at the time of initial diagnosis (ID) and relapse. Notably, the expression of CD47 did neither differ for bulk, nor for AML LSCs, between the two disease states (Figure 1D). MFI ratios of CD47 expression on bulk AML cells corresponded to 48.67 at ID and 50.04 after relapse, and 32.31 and 32.01 on AML LSCs (ID and relapse, respectively).

Collectively, these results confirm that CD47 is highly expressed on both bulk AML cells and AML LSCs. Most interestingly, CD47 expression in AML patients appears to be independent of the disease state.

### Generation and characterization of licMABs

**Figure 2:**
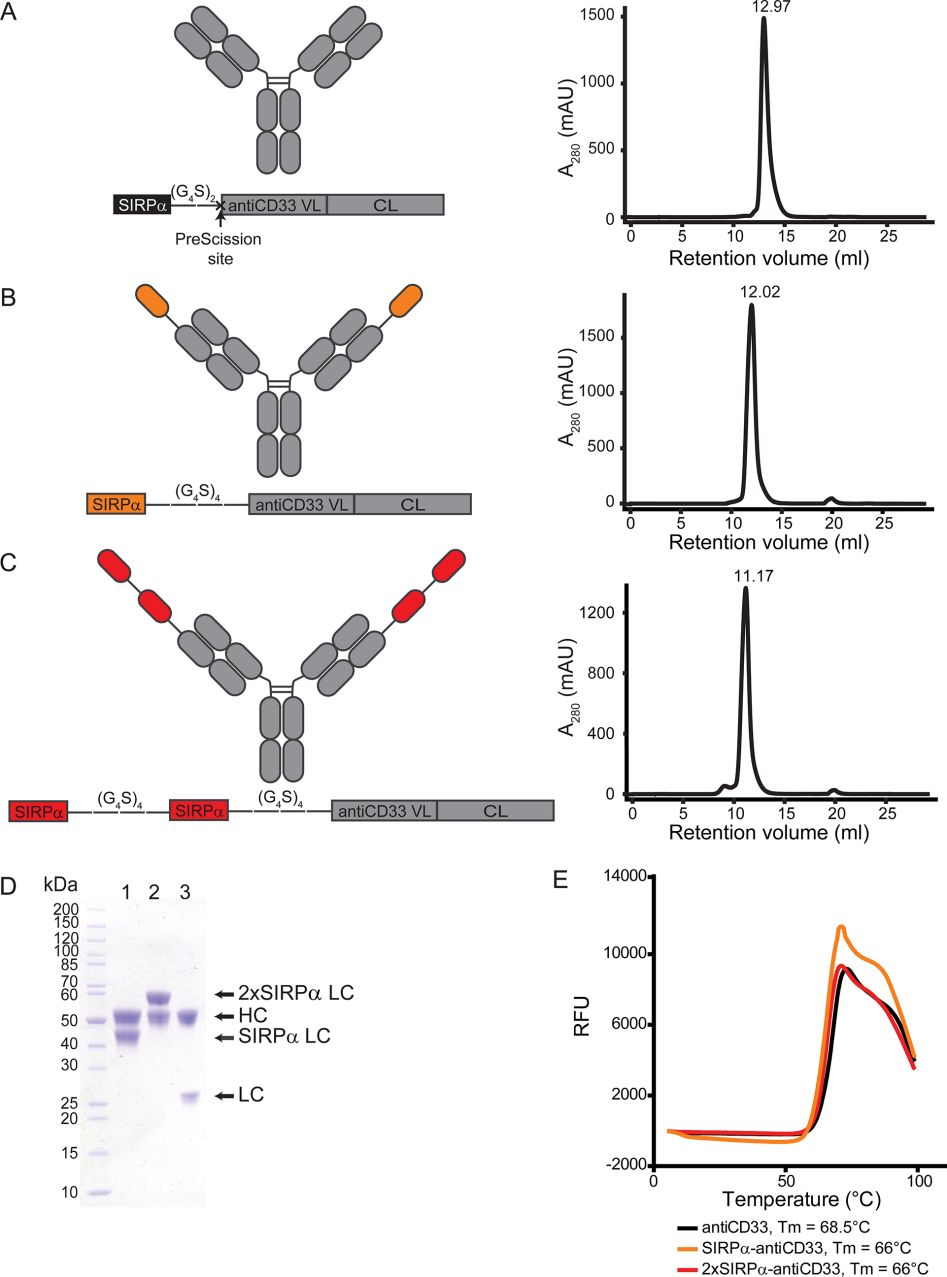
Generation and characterization of licMAB molecules (**A**–**C**, left) Schematic representation of antiCD33 mAb, SIRPα-antiCD33 licMAB, 2xSIRPα-antiCD33 licMAB and the corresponding light chain engineered vectors (VL, variable light chain; CL, constant light chain). (A-C, right) Size exclusion chromatography analyses of the licMABs and mAb. (**D**) SDS-page analysis of purified SIRPα-antiCD33 licMAB (1), 2xSIRPα-antiCD33 licMAB (2) and antiCD33 mAb (3) under reducing conditions. Bands corresponding to the heavy chain (HC) or light chain (LC) of each molecule are indicated. (**E**) Melting curves of antiCD33 mAb, SIRPα-antiCD33 licMAB and 2xSIRPα-antiCD33 licMAB, determined by fluorescence thermal shift (RFU, Relative Fluorescence Units). The measured melting points (Tm) of each molecule are specified.

To locally interfere with the CD47-SIRPα axis at the tumor site, we generated the so-called local inhibitory checkpoint monoclonal antibodies (licMABs), which consist of the endogenous SIRPα V-like domain linked to a mAb targeting CD33, a validated target antigen in AML [29] (Figure 2). The variable fragment of the antiCD33 antibody clone hP67.7 was grafted onto an IgG1 scaffold and served as basis for generating licMABs. To create the SIRPα-antiCD33 licMAB, the N-terminal Ig V-like domain of SIRPα was linked by a flexible polyglycine-serine linker (G_4_S) of 4 repeats to the N-terminus of the antiCD33 light chain (Figure 2B). A second SIRPα domain was linked by a (G_4_S)_4_ linker to the N-terminus of the SIRPα-antiCD33 light chain to obtain the 2xSIRPα-antiCD33 licMAB (Figure 2C). For generation of the antiCD33 mAb, a PreScission protease cleavage site was inserted between the SIPRα domain and the antiCD33 light chain (Figure 2A).

SIRPα-antiCD33 licMAB, 2xSIRPα-antiCD33 licMAB and antiCD33 mAb were produced in Expi293F cells and purified by Protein A affinity chromatography with yields of 83.5, 67.5 and 31 mg/L of culture medium, respectively. Further purification of the proteins by size exclusion chromatography confirmed the proteins integrity with no significant aggregation, protein degradation or contamination (Figure 2A–2C). Two equimolar bands were visible in SDS-PAGE analysis for all molecules, corresponding to the computed masses of 49.0 kDa for the heavy chain and 38.7, 53.3, and 24.0 kDa for the SIRPα, 2xSIRPα and antiCD33 light chain, respectively (Figure 2D). Further measurements by fluorescence thermal shift assays revealed a melting point of 68.5°C for the antiCD33 mAb, whereas the melting temperature was reduced to 66°C when the SIRPα domain was present (Figure 2E). Taken together, these results show that licMAB molecules can be produced with a high yield and purity and that they are stable at elevated temperatures.

### LicMABs bind to CD33 with high affinity and weakly interact with CD47

**Figure 3:**
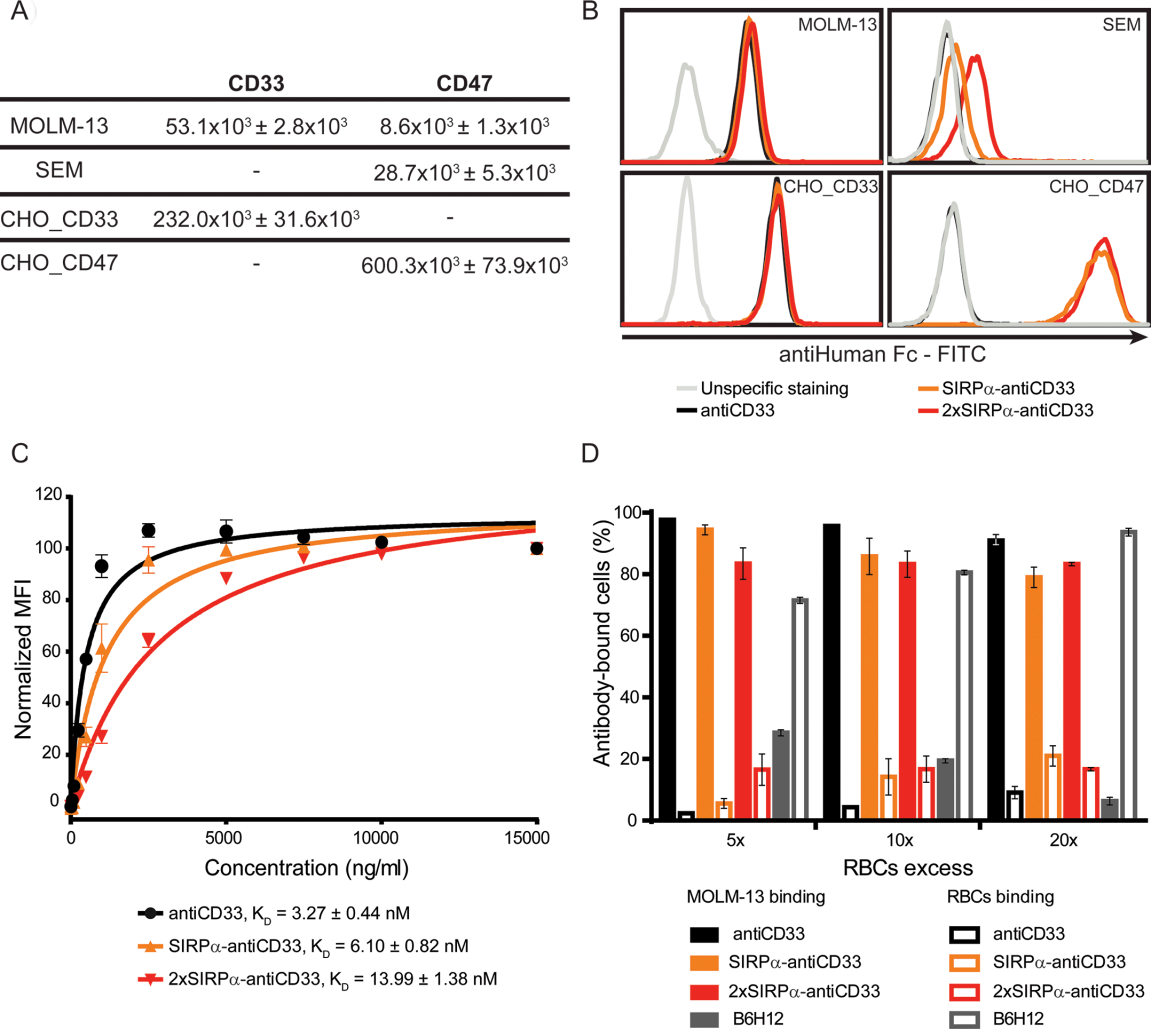
LicMABs bind strongly to CD33 and weakly to CD47 (**A**) Quantification of CD33 and CD47 antigens expressed on the cell surface of MOLM-13, SEM, CHO_CD33 and CHO_CD47 cell lines assessed with QIFIKIT. Data is displayed as the mean ± SEM of four independent experiments. (**B**) Binding analysis of antiCD33 mAb, SIRPα-antiCD33 licMAB and 2xSIRPα-antiCD33 licMAB to different cell lines by flow cytometry detected by a secondary FITC-conjugated antiHuman Fc antibody. The grey line indicates unspecific staining of the secondary antibody to the corresponding cells. The experiment was performed three times with similar results. (**C**) K_D_ determination of antiCD33 mAb, SIRPα-antiCD33 licMAB and 2xSIRPα-antiCD33 licMAB on MOLM-13 cells measured by calibrated flow cytometry. Mean values and SEM (error bars) of three independent experiments are plotted and K_D_ values are indicated. (**D**) Percentage of MOLM-13 cells or RBCs within the antibody-bound cells. Error bars indicate SEM of three independent experiments with three different donors.

To evaluate the ability of licMABs to bind to CD33 and CD47 antigens, four different cell lines were used. The CD33- and CD47-expressing AML cell line MOLM-13 was the main cell line used in this work. SEM, a CD33 negative, CD47 positive B-lineage Acute Lymphoid Leukemia (ALL) cell line, was used to study SIRPα-CD47 interactions independent of CD33 binding. Flp-IN^TM^-CHO cells were stably transfected to generate CD33 or CD47 single positive cell lines (here designated as CHO_CD33 and CHO_CD47) and were used as a control for the binding studies. Notably, CD33 and CD47 expression levels differ between these cell lines (Figure 3A). CD47 is expressed 3.3-fold higher on SEM cells and 68.8-fold higher on CHO_CD47 cells than on MOLM-13 cells, and CD33 expression is 4.4-fold higher on CHO_CD33 than on MOLM-13 cells.

All molecules bound comparably to the CD33 and CD47 double positive MOLM-13 cell line, irrespective of the presence of the SIRPα domain (Figure 3B). In addition, a comparable staining intensity was detected for all molecules on CHO_CD33 cells, indicating that the SIRPα domain does not interfere with CD33 recognition and binding. Since SEM cells do not express CD33, no binding was observed for the antiCD33 mAb. However, we detected a minimal staining signal for the SIRPα-antiCD33 licMAB (MFI ratio = 1.5) on SEM cells, which was increased for 2xSIRPα-antiCD33 licMAB (MFI ratio = 2.81), in comparison to the antiCD33 mAb (MFI ratio = 1). This result is due to a weak binding of the SIRPα domain to CD47 expressed on SEM cells (Figure 3B). To assess the binding capability of the single and tandem SIRPα domains, we used the engineered CHO_CD47 cell line. Presumably because of the very high surface expression of CD47, both licMABs showed an identical staining pattern on CHO_CD47 cells. Consequently, the strength of CD47 binding positively correlates with the quantity of SIRPα domains present within the licMAB and the numbers of CD47 molecules present on the target cell surface.

To quantitatively compare the binding strength of each molecule to MOLM-13 cells, we determined K_D_ values by avidity measurements using calibrated flow cytometry. SIRPα-antiCD33, 2xSIRPα-antiCD33 and antiCD33 antibodies showed affinity values in the low nM range, consistent with other CD33-targeting agents (Figure 3C) [21, 24, 25]. Moreover, the similarity of the K_D_ values obtained for all three molecules confirmed that tumor antigen-binding is not strongly influenced by the presence of the SIRPα domain or its interaction with CD47. The affinity of SIRPα for CD47 could not be quantified by flow cytometry due to the weak CD47-SIRPα interaction. However, μM affinities between CD47 and SIRPα have been described in previous studies [15, 16].

We hypothesized that the high affinity for CD33 of the licMABs would facilitate preferred tumor antigen-binding over healthy CD47-expressing cells. RBCs, which express CD47 [7], are a likely antigen sink as they are highly abundant and accessible in the bloodstream. To test whether licMABs selectively bind CD33 and CD47 double positive cells in the presence of RBCs, MOLM-13 cells were mixed with a 5-, 10- or 20-fold excess of RBCs and analyzed for licMAB binding by flow cytometry. To exclude that the binding ability of licMABs is resulted from the fact that MOLM-13 express Fc receptors on their surface and RBCs are Fc receptor free, a human IgG1 isotype control was included in the assay. The human IgG1 isotype control did neither bind to MOLM-13 cells nor RBCs (Supplementary Figure S1), whereas the antiCD33 mAb and licMABs preferentially bound to MOLM-13 cells even in the excess of RBCs. Interestingly, the presence of a second SIRPα domain on the licMAB did not influence the binding to RBCs compared to the SIRPα-antiCD33 licMAB (Figure 3D). A high affinity antiCD47 antibody (clone B6H12), which served as a control favorably bound to RBCs in all conditions. These results confirm our hypothesis that licMAB binding is dictated by the high affinity targeting to CD33 and not influenced by the presence of CD47 on healthy cells.

### LicMABs internalize upon bivalent CD33 binding

**Figure 4:**
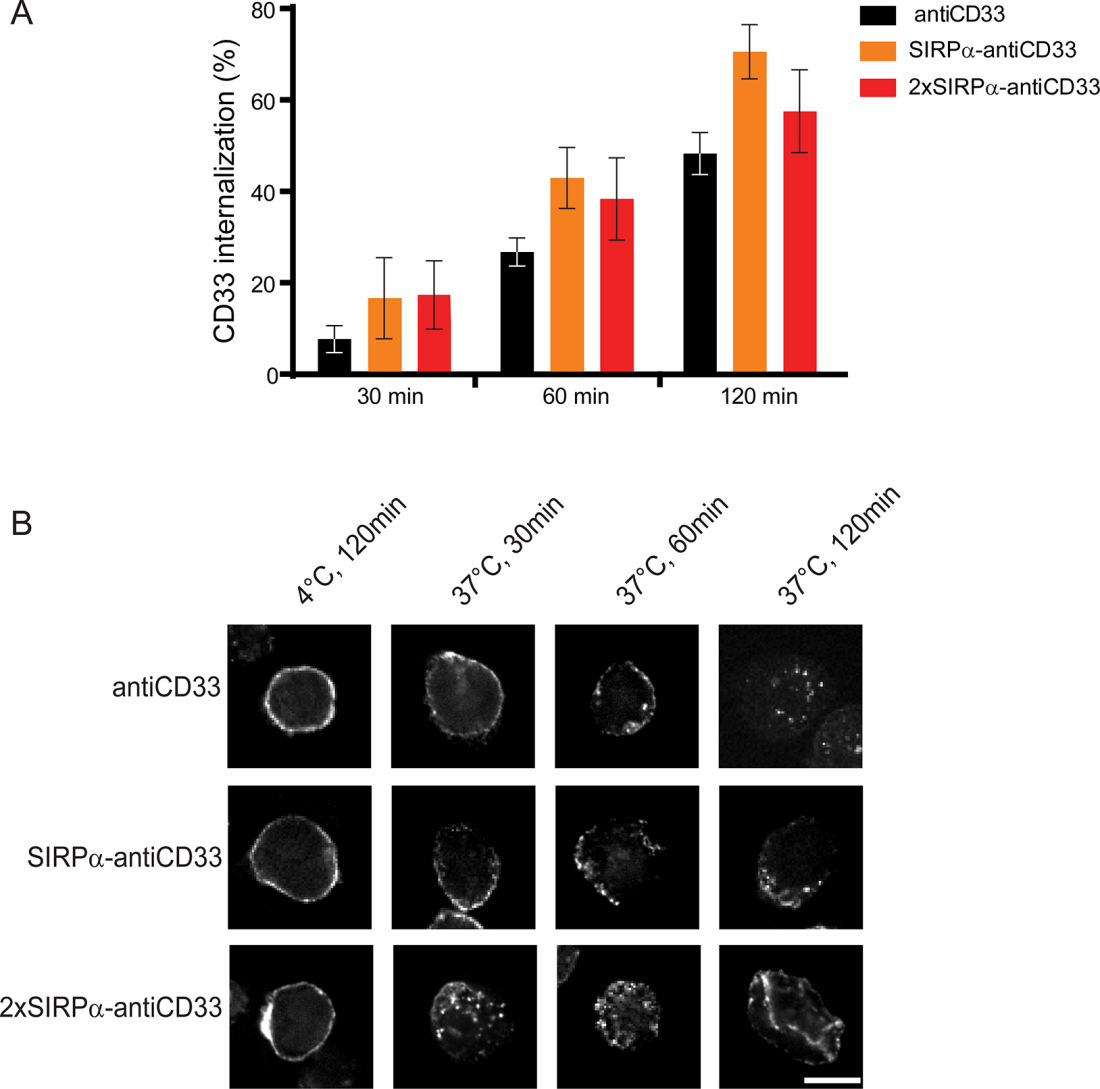
LicMAB molecules internalize upon CD33 binding (**A**) Internalization of antiCD33 mAb, SIRPα-antiCD33 licMAB and 2xSIRPα-antiCD33 licMAB on MOLM-13 cells assessed by flow cytometry. Percentage of internalized molecules relative to cells kept on ice water of three independent experiments is shown (error bars indicate SEM). (**B**) Directly labeled antiCD33 mAb, SIRPα-antiCD33 licMAB and 2xSIRPα-antiCD33 licMAB were incubated with MOLM-13 cells either at 4°C or at 37°C for 30, 60 or 120 min and internalization was visualized by confocal microscopy (scale bar = 10 μm).

Since bivalent mAbs against CD33, and specifically the antiCD33 antibody clone P67.6, were shown to internalize upon cross-linking [30–32], we tested the CD33-targeting licMABs for internalization. To this end, CD33 and CD47 double positive MOLM-13 cells were incubated with licMABs and mAb and internalization was determined by flow cytometry based on the signal of the secondary antibody. SIRPα-antiCD33 and 2xSIRPα-antiCD33 licMABs, as well as the antiCD33 mAb, exhibited a similar internalization rate, increasing over time (Figure 4A). 14.8 ± 3.1% of the molecules were internalized after 30 min of incubation, 37.0 ± 3.1% after 60 min and 58.8 ± 3.5% after 120 min, which is in agreement with data reported previously [31, 32].

To confirm the results obtained by flow cytometry, confocal microscopy using directly labeled licMABs or mAbs was performed. Confocal images showed a clear membrane-bound staining for control samples incubated at 4°C, where internalization does not take place (Figure 4B). However, a re-localization of the molecules was observed when the sample was incubated at 37°C. After 30 and 60 min of incubation, SIRPα-antiCD33 licMAB, 2xSIRPα-antiCD33 licMAB and antiCD33 mAb staining appeared on both the cell membrane and intracellular sites and after 120 min only low levels of intracellular signal were detected due to degradation of the molecules or bleaching of the coupled dye in low-pH lysosomal compartments. In summary, 2xSIRPα-antiCD33 and SIRPα-antiCD33 licMABs, as well as antiCD33 mAb, displayed similar internalization rates.

### LicMABs mediate specific lysis of AML cell lines

One of the main mechanisms by which IgG1 antibodies induce the elimination of antibody-bound cells is ADCC [2]. Therefore, we hypothesized that licMABs (engineered from an IgG1 antibody) would induce ADCC of antigen-expressing cells. To test this, calcein-labeled MOLM-13 cells were co-incubated with freshly isolated NK cells in the presence of increasing licMAB concentrations.

**Figure 5:**
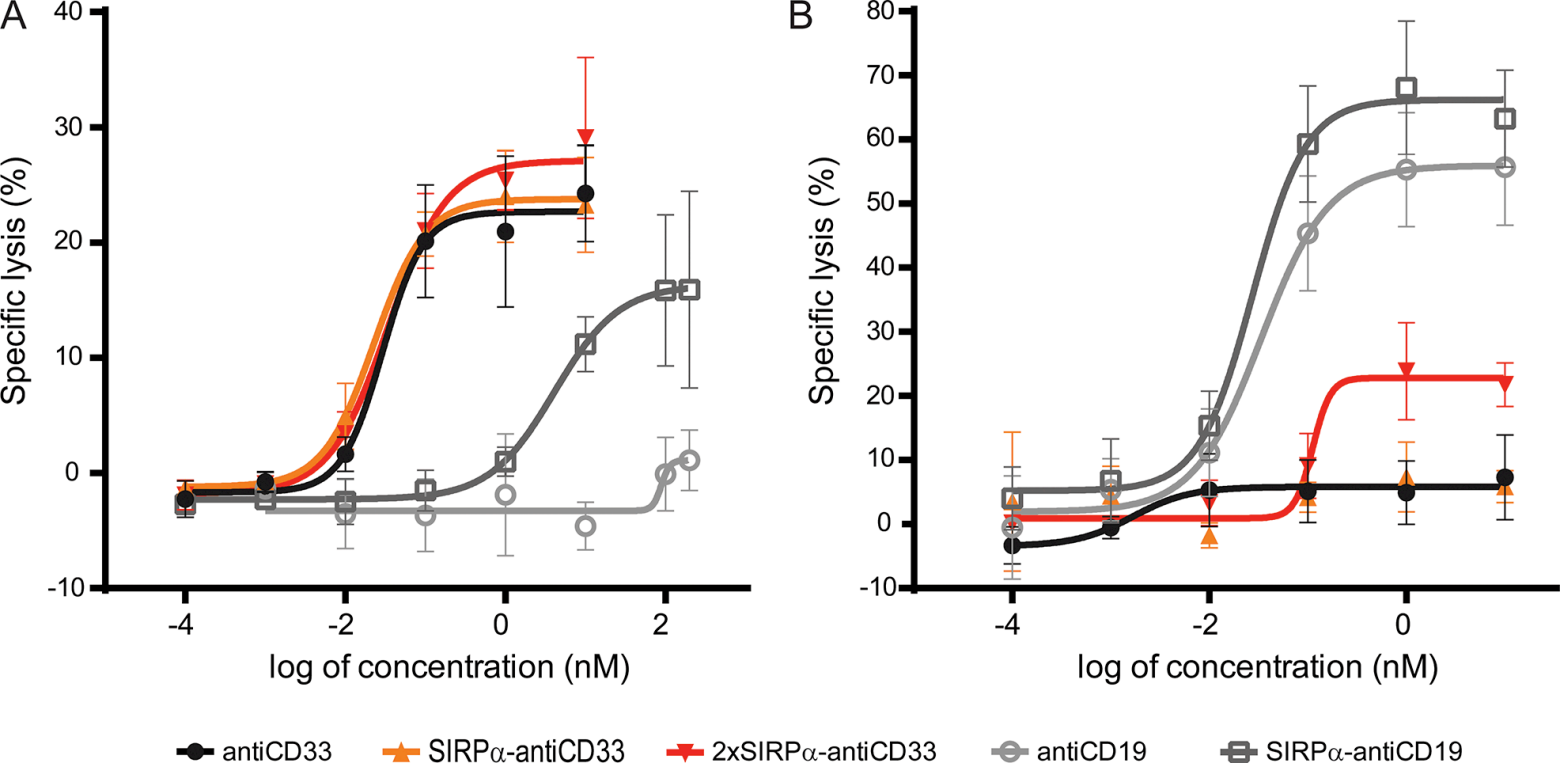
Specific cytotoxicity of tumor cells is mediated by licMABs Cytotoxic effects on MOLM-13 cells (**A**) and SEM cells (**B**) induced by antiCD33 mAb, SIRPα-antiCD33 licMAB, 2xSIRPα-antiCD33 licMAB, antiCD19 mAb and SIRPα-antiCD19 licMAB were analyzed by calcein release assays and plotted as a dose-response curve (*n* = 4, error bars = SEM).

SIRPα-antiCD33 and 2xSIRPα-antiCD33 licMABs, as well as antiCD33 mAb, efficiently and dose-dependently stimulated cytotoxicity of MOLM-13 cells achieving a maximum specific lysis of 20% at concentrations of 1 nM (Figure 5A). The rather low maximal specific lysis for this particular cell line is consistent with other ADCC studies on MOLM-13 cells [27, 33]. As a control molecule, we used a human IgG1 mAb targeting CD19, a B-cell lymphoma antigen, which did not induce killing of MOLM-13 cells due to the lack of CD19 expression. However, SIRPα-antiCD19 licMAB induced cell lysis at high concentrations (100 nM), probably due to minimal binding of SIRPα to CD47 expressed on MOLM-13 cells (Figure 5A). Nonetheless, the EC_50_ value of the CD19-directed licMAB (4 nM) was 100-fold higher than the EC_50_ value of the CD33-targeting licMABs (30 pM), clearly underlining antigen-specific cytotoxicity (Table 1).

**Table 1:** EC_50_ values (pM) obtained for licMABs and mAbs in ADCC assays with MOLM-13 and SEM cell lines as target cells

Molecule	MOLM-13	SEM
antiCD33 mAb	29.13	n.d.
SIRPα-antiCD33 licMAB	23.0	n.d.
2xSIRPα-antiCD33 licMAB	34.3	111.5
antiCD19 mAb	n.d.	33.4
SIRPα-antiCD19 licMAB	4089.0	27.4

n.d., not determined

Next, we investigated the cytotoxic effect of the SIRPα domain within the CD33-targeting licMABs by ADCC assays using the SEM cell line. SEM cells are derived from B-lineage ALL cells and are thus positive for CD19 and CD47 but negative for CD33. As effector cells, IL-2 expanded NK cells were used. Neither the control antiCD33 mAb nor the SIRPα-antiCD33 licMAB stimulated killing of the SEM cell line, demonstrating that NK cells were not recruited to CD47 positive cells by the SIRPα domain of the licMAB (Figure 5B). The 2xSIRPα-antiCD33 licMAB showed a minor specific lysis at 1 nM, indicating a CD47-targeting effect due to increased SIRPα quantities. As expected, SIRPα-antiCD19 licMAB and antiCD19 mAb induced specific killing of CD19 positive SEM cells, reaching maximal specific lysis of 65% at concentrations of 1 nM.

We have demonstrated the capability of licMABs to induce NK cell-mediated killing of target cells. However, CD33 is not only expressed on AML cells, but also on healthy cells from the myeloid lineage [34]. Thus, licMABs could potentially be detrimental to healthy cells co-expressing CD33 and CD47. Since CD33 expression has been described to be much higher on AML cells compared to healthy cells [29], we evaluated the ability of licMABs to preferentially target cells expressing high levels of CD33 in the presence of low CD33-expressing cells. We performed an ADCC assay using a 1:1 mixture of MOLM-13 and OCI-AML3 cells, the latter known to express lower levels of CD33. LicMABs and antiCD33 mAb preferentially induced lysis of MOLM-13 cells in comparison to OCI-AML3 cells at both concentrations evaluated (10 μM and at the EC_50_), indicating that cells expressing high levels of CD33, such as tumor cells, are preferentially cleared (Supplementary Figure S2).

Taken together, these results confirm that licMABs recruit and activate NK cells upon antigen binding. Furthermore, they imply that the endogenous low affinity extracellular domain of SIRPα does not function as a robust CD47-targeting agent by itself and that the activity of licMABs is dominated by high affinity binding to the tumor antigen.

### Phagocytosis of AML cell lines is enhanced by licMABs

The key goal of the engineered licMABs is to inhibit the CD47-SIRPα signaling pathway and thus increase the phagocytosis of tumor antigen-expressing cells. We reasoned that licMABs stimulate phagocytosis by delivering an Fc-mediated pro-phagocytic signal to FcRs on macrophages and simultaneously disrupt the native CD47-SIRPα interaction. To address this experimentally, we performed an antibody-dependent cellular phagocytosis (ADCP) assay with Macrophage-Colony Stimulator Factor (M-CSF) differentiated macrophages and MOLM-13 cells by Imaging Flow Cytometry (IFC).

To test the engulfment ability of the M-CSF differentiated macrophages, carboxylate microspheres were used instead of target cells, achieving a phagocytosis of 17.8 ± 2.3%. For negative controls, the ADCP assay was performed either at 4°C, where phagocytosis is prevented, or in presence of cytochalasin D, an inhibitor of actin polymerization [35]. As expected, phagocytosis was prohibited under control conditions (0.6 ± 0.1% and 1.0 ± 0.3% when incubated at 4°C or with cytochalasin D, respectively).

**Figure 6:**
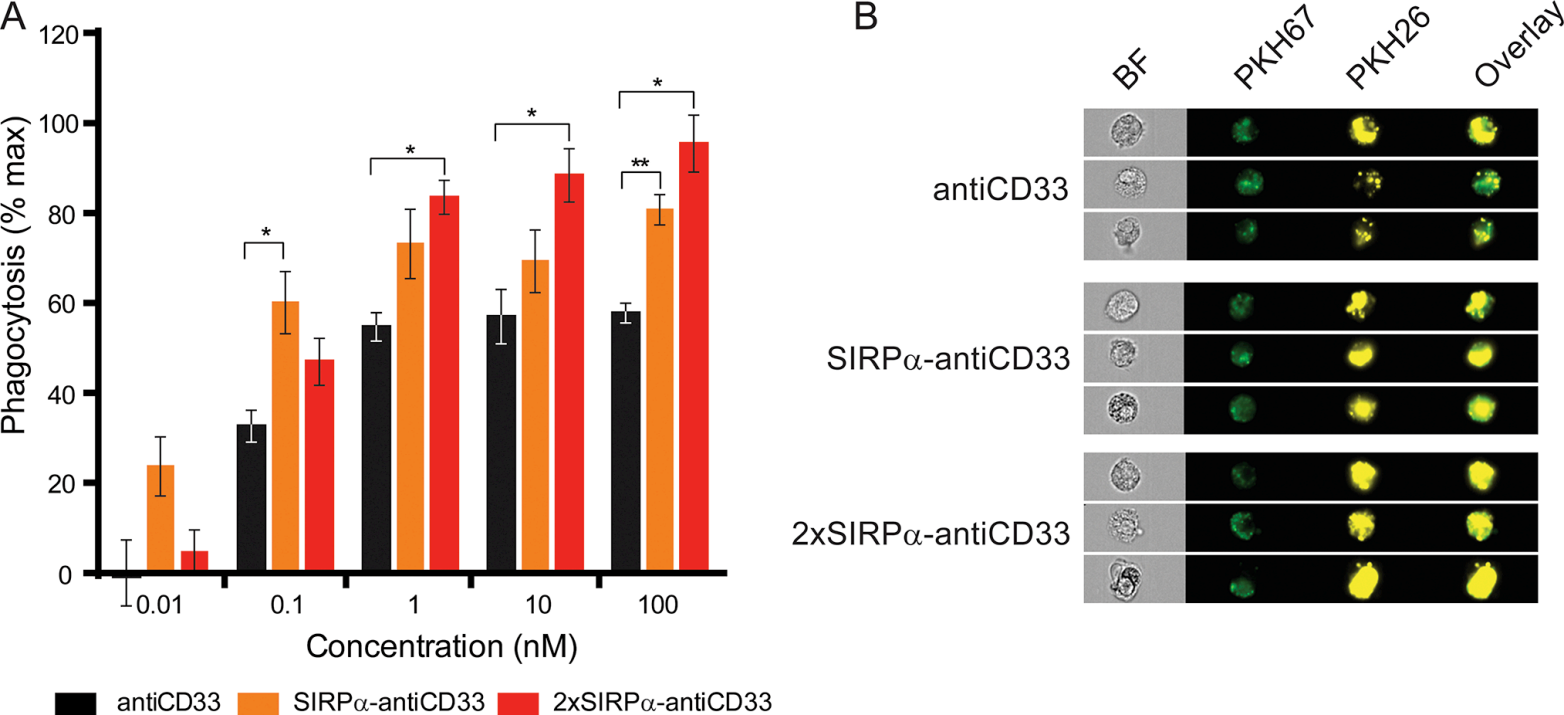
LicMAB molecules enhanced phagocytosis of MOLM-13 cells (**A**) Phagocytosis of MOLM-13 cells stimulated by antiCD33 mAb, SIRPα-antiCD33 licMAB and 2xSIRPα-antiCD33 licMAB at different concentrations was evaluated by imaging flow cytometry. Error bars indicate the SEM of three independent experiments using three different donors and statistical significance was calculated with a *t-test* with Welch's correction (*p-value* < 0.05*, < 0.01**). (**B**) Representative images of PKH67 (macrophages) and PKH26 (MOLM-13) positive single cells that were defined as phagocytic events obtained by imaging flow cytometry (BF, bright field).

Importantly, the addition of the antiCD33 licMABs and mAb promoted phagocytosis of MOLM-13 cells in a concentration-dependent manner (Figure 6A). Phagocytosis was significantly enhanced by blocking the CD47-SIRPα axis, with respect to the control antiCD33 antibody, for the 2xSIRPα-antiCD33 licMAB at concentrations higher than 0.1 nM and for the SIRPα-antiCD33 licMAB at 0.1 nM and 100 nM (Figure 6A, 6B). The ability of licMABs to enhance phagocytosis of AML cell lines was confirmed using another CD33 and CD47 positive AML cell line (OCI-AML3) as target cells (Supplementary Figure S3).

In summary, these results suggest that licMABs interfere with the CD47-SIRPα axis upon tumor antigen binding, thus blocking the inhibitory signal in macrophages and enhancing specific phagocytosis of target AML cell lines.

### LicMABs efficiently promote elimination of primary, patient-derived AML cells

After determining the functionality of licMABs to mediate killing and phagocytosis of established AML cell lines, we sought to evaluate the cytotoxicity of these molecules in primary patient-derived AML cells in a non-autologous setting. For that purpose, primary NK cells from healthy donors were co-incubated for 24 h *ex vivo* with AML patient cells and licMABs and analyzed by flow cytometry.

**Figure 7:**
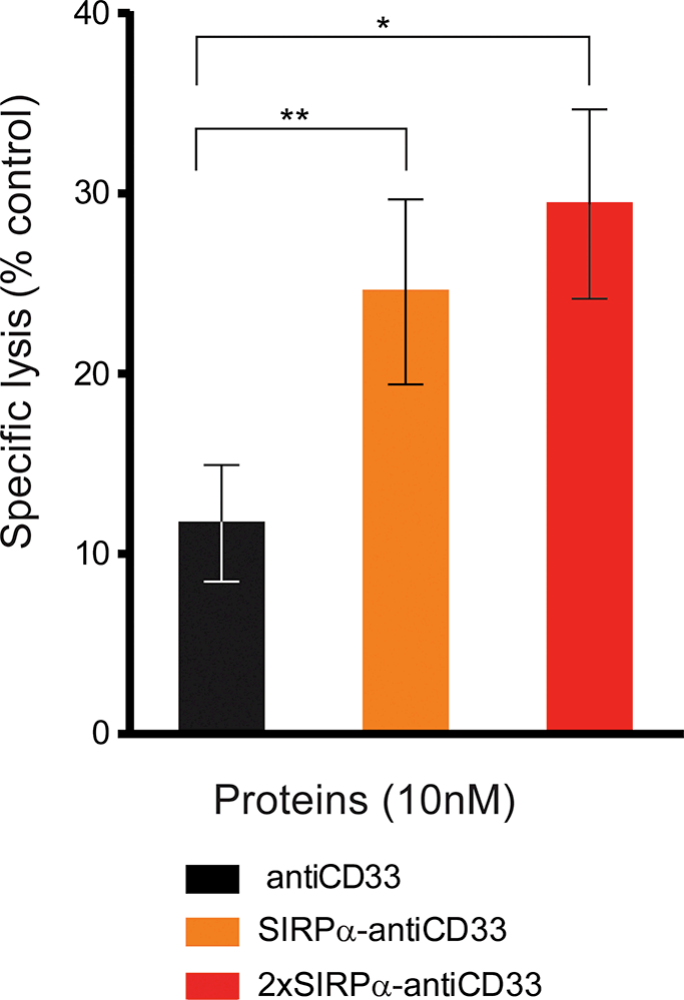
LicMABs induced NK cell-mediated cytotoxicity of AML patient samples Cytotoxicity of primary AML patient cells triggered by antiCD33 mAb, SIRPα-antiCD33 licMAB and 2xSIRPα-antiCD33 licMAB at a concentration of 10 nM was analyzed by determining the percentage of remaining CD33 or CD123 positive cells by flow cytometry. Error bars indicate the SEM of 9 different AML patient samples. Statistical differences were assessed using the Wilcoxon test (*p-value* < 0.05*, < 0.01**).

SIRPα-antiCD33 and 2xSIRPα-antiCD33 licMABs successfully triggered NK cell-mediated cytotoxicity of AML patient cells. Notably, licMAB molecules induced significantly improved killing of AML cells compared with antiCD33 mAb, presumably due to avidity binding of SIRPα domains (Figure 7). Although there was a clear overall tendency in the response of analyzed patient samples, individual differences were observed, demonstrating a relevant degree in heterogeneity between AML patients (Supplementary Figure S4). Interestingly, patients 4 and 6 extremely benefited from licMABs antitumor activity, achieving a 4.1- and 38.4-fold elevated specific lysis of primary AML cells, respectively, in comparison to the control antiCD33 mAb. Furthermore, patients 7 and 9 profited from the second SIRPα domain by obtaining a 2.5- and 4.2-fold increased specific lysis, respectively, with respect to the SIRPα-antiCD33 licMAB.

Taken together, these results show that licMABs efficiently induce NK cell-mediated killing of primary AML patient cells *ex vivo*.

## DISCUSSION

In this study we report the development of licMABs, novel therapeutic agents that combine specific tumor antigen binding, Fc-mediated immune cell recruitment and activation, and simultaneous disruption of the CD47-SIRPα axis. LicMABs, generated by grafting the endogenous N-terminal extracellular domain of SIRPα onto an IgG1 antibody targeting the AML antigen CD33, successfully induced NK cell-mediated killing of AML cell lines and primary AML patient cells and enhanced phagocytosis of AML cell lines. The antitumor activity triggered by these molecules identifies them as a promising therapeutic approach for AML.

Previous studies analyzed CD47 expression on AML cells by comparing its expression levels on AML LSCs with respect to hematopoietic stem cells (HSCs) from healthy donors and concluded that CD47 is overexpressed on AML LSCs [8]. Here, we further characterized CD47 expression levels on AML patient samples and demonstrated that CD47 is highly expressed on both the bulk AML cells and AML LSCs. Moreover, CD47 expression on AML cells did not differ between initial diagnosis of the disease or at time of relapse. These results imply that therapies that block the CD47-SIRPα axis, as licMABs, target bulk AML cells as well as AML LSCs. Most importantly, such therapies would thus be suited for not only initially diagnosed, but also relapsed patients, for whom there is no current standard treatment [36].

The blockade of the CD47-SIRPα signaling pathway has recently been investigated as a therapeutic tool, and several approaches to disrupt the CD47-SIRPα axis, including mAbs against CD47 and CD47-antagonists, have been shown to stimulate clearance of tumor cells by phagocytosis [9–13, 17, 37]. However, due to the ubiquitous expression of CD47, high affinity binding agents may cause undesired toxicity to healthy cells. Furthermore, cells expressing CD47, such as RBCs, might function as an antigen sink and could prevent CD47-targeting molecules from binding to tumor cells *in vivo*. To avoid direct targeting of CD47 but still interfere with the CD47-SIRPα signaling pathway, we took advantage of the naturally occurring weak interaction between CD47 and SIRPα. We therefore hypothesized that the engraftment of the endogenous SIRPα domain, which has a low affinity towards CD47 [16], onto a mAb targeting CD33 with high affinity, would result in a molecule that specifically recognizes and binds to AML cells via CD33 and locally blocks the CD47-SIRPα pathway. The resulting licMAB would induce killing and phagocytosis of tumor cells while avoiding systemic side effects.

A similar approach was recently published, and the respective SIRPabodies, which combine an antiCD20 mAb and the endogenous SIRPα domain, were shown to bind CD20 and CD47 on dual expressing cells, reduce tumor burden and prolong survival in xenograft lymphoma mouse models [17]. In this case, a single SIRPα extracellular domain was fused to the IgG1 heavy chain N- or C-terminal, whereas in the case of licMABs, a single or tandem SIRPα domain was fused to the N-terminus of the light chain. The fusion of the extracellular SIPRα domain to the N-terminus of the light or heavy chain generates a native N-terminus of the SIRPα part, but may interfere with the Complementarity-Determining Regions (CDRs) of the parental antibody. Alternatively, the fusion of the SIRPα domain to the C-terminus of the light or heavy chain most likely does not compromise the antibody's CDRs, but might impact CD47 binding. Therefore, the position of the SIRPα domain on SIRPα-antibody fusion proteins needs to be further optimized in order to achieve best antitumor effects.

Binding analyses performed in the present study demonstrate that licMABs bind to CD33 with high affinity and, consistent with our hypothesis, interact weakly with cells expressing CD47 but not CD33. This binding to CD47, however, correlated with surface expression of this molecule. Since CD47 is overexpressed on cancer cells with respect to normal cells, we propose that the SIRPα domain of the licMAB would not bind to normal cells expressing CD47 but facilitate the targeting of dual antigen-expressing tumor cells led by high affinity binding to CD33. This specific feature is of utmost importance in the context of circulating RBCs expressing CD47, which provide a large antigen sink and a high risk for toxicity as CD47 expression prevents clearance of RBCs *in vivo* [38]. Consequently, the binding properties of licMABs enable them to selectively bind to CD33-expressing tumor cells in the presence of excess of RBCs expressing CD47, thus overcoming the antigen sink that RBCs may represent. Additional binding analyses underline the hypothesis that the blockade of the CD47-SIRPα axis can be modulated by varying the amount of SIRPα domains in a licMAB. Taken together, the binding properties of licMABs enable these molecules to discriminate between CD33 negative and CD47 positive healthy cells, and CD33 and CD47 double positive tumor cells, and to selectively bind to the latter, avoiding undesired side effects.

Moreover, based on preferential lysis studies, we suggest that licMABs are preferentially directed to cells expressing high levels of CD33 on their surface, even in the presence of low CD33-expressing cells, which is in agreement with previous findings. For instance, a clear relation between CD33 expression and the efficacy of Gemtuzumab Ozogamicin (GO, a drug-conjugated antibody targeting CD33) was shown *in vitro* and *in vivo*, indicating that a higher cytotoxicity is correlated with higher numbers of CD33 expressed on target cells [32, 39]. Furthermore, a study that characterized a CD33-CD3 BiTE molecule (AMG 330) has demonstrated preferential lysis of cells with high CD33 expression compared to cells with low expression of CD33 [29]. These evidences support our hypothesis that licMABs target and induce clearance of high CD33 expressing cells, such as AML cells, and only have a mild effect on healthy cells expressing lower levels of CD33.

Even though blockade of the CD47-SIRPα axis interferes with inhibition of phagocytosis, studies using an engineered high affinity SIRPα domain, which functions as a CD47-antagonist. They demonstrated that an Fc-mediated pro-phagocytic stimulus is required in order to induce active phagocytosis of tumor cells [37]. Along similar lines, disruption of the CD47-SIRPα axis has been shown to synergize with the Fc-mediated pro-phagocytic stimulus of a mAb targeting a tumor antigen, such as rituximab, an antiCD20 mAb [12]. In order to benefit from the combination of the blockade of the CD47-SIRPα axis and a Fc-mediated stimulus in one single molecule, a bispecific antibody targeting CD20 and CD47 was generated [13]. CD20/CD47 bispecific antibodies reduced lymphoma burden and extended survival of NHL-engrafted mice, hence recapitulating the synergistic effects of antiCD47 and antiCD20 combination therapy. The results obtained with licMABs in this work are in agreement with those achieved by Piccione and coworkers [13, 17]. LicMAB molecules promote tumor specific phagocytosis by combining two pro-phagocytic signals: (1) blockade of the CD47-SIRPα signaling pathway by the endogenous SIRPα domain and (2) engagement of the FcRs by the Fc domain. Thus, we demonstrate that licMABs significantly enhanced phagocytosis of tumor cells with respect to the antiCD33 mAb.

Additionally, we proved that the IgG1 Fc domain of the licMAB is able to recruit and activate NK cells, therefore inducing cytotoxicity to targeted cells. Although unintended targeting to CD47 single positive cells might be supported by increased concentrations of licMABs or by attaching additional SIRPα domains, our results suggest a wide “therapeutic window”, i.e. a concentration range, where licMABs strongly favor elimination of tumor antigen-expressing cells and do not compromise cells that do not express the targeted tumor antigen. We consequently argue that with this approach, an undesired binding to CD47 expressed on healthy cells can be substantially reduced, lowering possible cytotoxic side effects. Studies with primary, patient-derived AML cells demonstrated that licMABs efficiently recruit and activate NK cells, thus inducing clearance of AML patient cells *ex vivo*. Notably, licMABs enhanced cytotoxicity of patient-derived AML cells with respect to the antiCD33 mAb in 7 out of 9 samples evaluated. Consistent with our hypothesis, increasing the local SIRPα concentration by adding a second SIRPα Ig V-like domain on the licMAB was beneficial for some patients. The favorable effects of the second SIRPα domain, however, cannot be generalized due to heterogeneity within patient samples. Hence, the optimal licMAB format has to be determined for each patient individually.

Besides the pro-phagocytic stimulus and NK cell cytotoxicity delivered by the Fc domain of the licMAB, we predict an additional tumor clearance effect by adaptive immune cells. After tumor cell phagocytosis, antigen-presenting cells (APCs), such as macrophages and dendritic cells (DCs), present tumor antigens to T cells triggering an adaptive immune response [40]. Therefore, stimulation of tumor cell phagocytosis by blocking the CD47-SIRPα axis enhances antigen-presentation and ultimately leads to increased tumor elimination [41, 42]. This hypothesis is supported by previous studies showing that an antiCD47 mAb induces cross-priming of CD8 positive T cells by macrophages and DCs, which results in effective antitumor T cell responses [41, 43].

A minor drawback of the CD33-targeting licMABs is the CD33-dependent internalization, which was previously described for other CD33-targeting antibody derivatives [31, 32]. Antibody-drug conjugated molecules targeting CD33, such as Gemtuzumab ozogamicin (GO) or SGN-CD33A have been developed to profit from CD33 internalization in order to deliver a drug into the tumor cell [24, 31]. Since the mode of action of licMAB molecules is based on the recruitment of immune effector cells, their internalization might hamper the immune response against the target tumor cell. Although licMABs induced cytotoxicity and phagocytosis of CD33 positive tumor cells, antitumor immune responses might be even greater if internalization would not occur. As no CD33-dependent internalization has been reported for monovalent agents, such as for AMG 330 [44], a strategy to reduce CD33-dependent internalization of licMABs is to target CD33 with only one binding site. Therefore, an antiCD33/SIRPα bispecific antibody, containing a single binding arm targeting CD33 and the endogenous N-terminal SIRPα domain on the other arm, has been developed and is currently under investigation. Although the resulting molecule would not retain the favorable avidity effects of a mAb and may therefore have a lower affinity towards CD33, monovalent targeting of CD33 might reduce internalization and this may enhance elimination of tumor cells. Furthermore, a trispecific molecule consisting of (1) a single chain variable fragment (scFv) binding to a tumor antigen, (2) the endogenous SIRPα domain to block the CD47-SIRPα signaling pathway and (3) a scFv to trigger recruitment and activation of effector cells may recapitulate the antitumor activity of SIRPα-antiCD33 licMAB and not induce CD33 internalization. Notably, this molecule would be smaller in size than the licMAB, which could be beneficial when transferring the CD47-SIRPα blockade to solid tumors, as tumor penetration appears to be a challenging issue. Another approach to avoid licMAB's internalization would be to target another antigen with reduced internalization, such as CD123, which is also a validated target antigen overexpressed on AML LSCs [45], and retains a very low internalization rate upon binding [46]. Taken together, CD33-dependent internalization of licMABs could be overcome by different strategies, which should be further explored in order to have a maximal effect on tumor clearance.

In summary, the novel therapeutic agents called licMABs promoted NK cell-mediated cytotoxicity on CD33 and CD47 double positive AML cell lines and primary, patient-derived AML cells and did not trigger depletion of CD33 negative, CD47 positive cells. Moreover, by combining the recruitment and activation of FcRs via the antibody Fc domain and the blockade of CD47-SIRPα interaction by the endogenous low affinity CD47-antagonistic SIRPα domain, licMAB molecules significantly enhanced phagocytosis of tumor cells. Collectively, licMAB's characteristics and antitumor properties establish these molecules as promising therapeutic tools for the treatment of AML.

## MATERIALS AND METHODS

### SIRPα-antiCD33 licMAB, 2xSIRPα-antiCD33 licMAB and antiCD33 mAb construction and production

The antiCD33 variable light (VL) and variable heavy (VH) (clone hP67.6) were generated using custom gene synthesis (GeneArt, Thermo Fisher Scientific). The antiCD33 VL was subcloned into the pFUSE2-CLIg-hk vector (InvivoGen) and the antiCD33 VH into the pFUSE-CHIg-hG1 vector (InvivoGen). To generate the SIRPα-antiCD33 licMAB, the N-terminal Ig-like V-type domain of SIRPα (residues 1-120) was synthesized using custom gene synthesis (GeneArt, Thermo Fisher Scientific) and subcloned into the N-terminus of the antiCD33 light chain (LC) together with a (G_4_S)_4_ linker. A second SIRPα-(G_4_S)_4_ linker cassette was cloned N-terminal of the SIRPα-antiCD33 LC to obtain the 2xSIRPα-antiCD33 light chain. A cassette of SIRPα-(G_4_S)_2_ linker, containing a PreScission protease cleavage site (PreSc) at the C-terminus, was cloned N-terminal of the antiCD33 LC to generate a SIRPα-PreSc-antiCD33 antibody with a cleavable SIRPα. The corresponding plasmids were transfected into Expi293F cells (Thermo Fisher Scientific) according to the manufacturer's protocol. After five to seven days, the cell culture supernatant was harvested and licMABs were purified by protein A affinity chromatography. To obtain the antiCD33 mAb, SIRPα-PreSc-antiCD33 was incubated with PreScission protease for 6 h followed by a second round of protein A affinity chromatography. LicMABs and mAb were dialyzed against Phosphate Buffered Saline (PBS) and size exclusion chromatography (SEC) of the purified molecules was performed using a Superdex 200 increase 10/300 column (GE Healthcare Life Sciences, Little Chalfont, Buckinghamshire, United Kingdom). LicMABs and mAb were then analyzed by 4–20 % SDS-PAGE (Expedeon) under reducing conditions and visualized by Coomassie Brilliant Blue staining. Protein concentration was measured with a spectrophotometer (NanoDrop, GE Healthcare Life Sciences, Little Chalfont, Buckinghamshire, United Kingdom) and aliquots were stored at –80°C.

### Cell lines

The MOLM-13 and OCI-AML3 cell lines were purchased from the ‘Deutsche Sammlung von Mikroorganismen und Zellkulturen’ (DSMZ, Leibniz-Institut DSMZ, Braunschweig, Germany), the SEM cell line from the American Type Culture Collection (ATCC, Rockville, MD, USA) and the Flp-IN^TM^-CHO cell line from Thermo Fisher Scientific (Waltham, Massachusetts, USA). MOLM-13 cells were cultured in RPMI 1640 + GlutaMAX (Gibco, Thermo Fisher Scientific) and supplemented with 20% fetal bovine serum (FBS, Gibco, Thermo Fisher Scientific). SEM and OCI-AML3 cell lines were grown in RMPI 1460 + GlutaMAX (Thermo Fisher Scientific) with 10% FBS. The Flp-INTM-CHO cell line was engineered to stably express human CD33 (CHO_CD33) or human CD47 (CHO_CD47) and maintained in selection media (Ham´s F-12 (Biochrom), 10% FBS and 550 μg/mL hygromycin B gold (InvivoGen)). The Expi293F cell line was obtained from Thermo Fisher Scientific and cultured in Expi293 Expression Medium.

### Patients

After written informed consent in accordance with the Declaration of Helsinki and approval by the Institutional Review Board of the Ludwig-Maximilians-Universität (Munich, Germany), peripheral blood (PB) or bone marrow (BM) samples were collected from healthy donors (HDs) and patients with AML at initial diagnosis or relapse. PB or BM samples from AML patients were cryoconserved at ≤ –80°C in 80% FCS and 20% dimethyl sulfoxide (Serva Electrophoresis) until usage. PB from HDs was obtained on the day of the experiment.

For CD47 expression analysis, PB or BM samples from AML patients (*n* = 213) at initial diagnosis (*n* = 182) or relapse (*n* = 31) were collected between January 2014 and May 2016. AML was diagnosed according to the FAB (French-American-British) classification by the Laboratory of Leukemia Diagnostics of the Department of Internal Medicine III of the Klinikum der Universität München [47].

### Preparation of RBCs, peripheral blood mononuclear cells (PBMCs), NK cells and monocytes from whole human blood

RBCs were isolated from HDs PB by centrifugation and subsequently washed with RBC's wash buffer, as described previously [48]. PBMCs from AML patients and HDs were separated from PB by density gradient using the Biocoll separating solution (Biochrom), according to the manufacturer's protocol. NK cells were either expanded *ex vivo* by culturing PBMCs under IL-2 stimulus as described previously [49] or freshly isolated by magnetic separation using a human NK cell isolation kit (MACS Miltenyi Biotech) according to the manufacturer's protocol. Monocytes were isolated from PBMCs by magnetic separation with human CD14 MicroBeads (MACS Miltenyi Biotech) following the manufacturer's instructions.

### Thermal stability

The thermal stability of the licMABs and mAb was determined by fluorescence thermal shift assays using the CFX96 Touch Real-Time PCR Detection System (Bio-Rad, Munich, Germany) [50]. 10 μg of protein containing 1x SYPRO Orange (Thermo Fisher Scientific) were measured using FAM and SYBR Green I filter pairs.

### Detection of binding by flow cytometry

If not otherwise stated, flow cytometry analyses were performed on a Guava easyCyte 6HT instrument (Merck Millipore, Billerica, Massachusetts, USA) and data was plotted with GuavaSoft software version 3.1.1 (Merck Millipore, Billerica, Massachusetts, USA).

MOLM-13, SEM, CHO_CD33 and CHO_CD47 cells were stained with 15 μg/ml of licMABs or mAb followed by staining with a secondary FITC antiHuman IgG Fc antibody (clone HP6017, BioLegend). The median fluorescence intensity (MFI) ratio was calculated dividing MFI of the antibody by the MFI of the isotype control.

### Quantitative determination of cell surface antigens

The QIFIKIT kit (Dako) was utilized to quantify antigen surface expression following the manufacturer's instructions [51]. Briefly, cells were incubated with saturating concentrations of primary mouse antibody against human CD33 or CD47 (clone P67.7, BioLegend and clone CC2C6, BioLegend, respectively). Cells and QIFIKIT beads were stained in parallel with polyclonal Goat antiMouse FITC-conjugated (QIFIKIT, DAKO) at saturating concentrations. The MFI was plotted to obtain a calibration curve, which was used to interpolate the MFI values of the samples and obtain the numbers of antigenic sites on the cells.

### K_D_ determination

CD33 equilibrium binding constants (K_D_, as an avidity measurement) of the licMABs and mAb were determined by calibrated flow cytometry analyses as previously described [52]. Briefly, MOLM-13 cells were incubated with licMABs or mAb in concentrations ranging from 0.01 to 15 μg/ml and stained with a FITC antiHuman IgG Fc (clone HP6017, BioLegend) secondary antibody by flow cytometry. The instrument was calibrated with 3.0–3.4 μm Rainbow Calibration particles of 8 peaks (BioLegend), the maximum MFI value was set to 100% and all data points were normalized accordingly. The assay was performed in quadruplicates and the values were analyzed by non-linear regression using a one-site specific binding model.

### RBCs competition assay

MOLM-13 were stained with PKH26 (Sigma-Aldrich) following the manufacturer's instructions. Next, labeled MOLM-13 cells were mixed with freshly isolated RBCs in 5-, 10- or 20-fold excess and incubated with 15 μg/ml of licMABs, antiCD33 mAb or antiCD47 (clone B6H12, eBioscience). Antibody binding was detected with a secondary FITC antiHuman IgG Fc antibody (clone HP6017, BioLegend) by flow cytometry. The MFI of antibody-bound cells (FITC positive) was obtained and the MFI ratio of the sample containing licMABs, mAbs or the IgG1 isotype control (clone ET901, Biolegend) divided by the MFI value of the control sample containing uniquely the secondary antibody was calculated. The percentage of MOLM-13 cells (PKH26 positive) or RBCs (PKH26 negative) within the antibody-bound cells was determined. Single replicates from 3 different donors were averaged together (mean ± SEM).

### Internalization assay by flow cytometry

MOLM-13 cells were incubated with 15 μg/ml of licMABs or mAb either on ice-cold water for 120 min (to prevent internalization) or at 37°C for 30, 60 or 120 min. Cells were then washed with ice-cold FACS buffer and antibodies remaining on the surface were detected by staining with FITC antiHuman IgG Fc (clone HP6017, BioLegend). To define the background fluorescence, MOLM-13 cells were directly stained with the secondary antibody. The internalization was calculated as follows:

$$\text{Internalization}(\%)=\frac{{(\text{MFI}}_{4{}^{\circ}\text{C}})-{\text{ MFI}}_{\text{background}})\;-{\;(\text{MFI}}_{37{}^{\circ}\text{C}})\;-{\text{ MFI}}_{\text{background}})}{{(\text{MFI}}_{4{}^{\circ}\text{C}}{\;-\text{ MFI}}_{\text{background}})}\times 100$$

### Internalization assay by confocal microscopy

MOLM-13 cells were cultured on a poly-L-lysine (Sigma-Aldrich) coated 96-well plate. Subsequently, cells were incubated with 15 μg/ml of licMABs or mAb directly labeled with Alexa Fluor 488 (Antibody Labeling Kit, Thermo Fisher Scientific), either on ice-cold water for 120 min or at 37°C for 30, 60 or 120 min. Then, cells were fixed and permeabilized in 20 mM PIPES pH 6.8, 4% formaldehyde, 0.2% Triton X-100, 10 mM EGTA, 1 mM MgCl2 at room temperature for 10 min, followed by incubation in blocking solution (3% bovine serum albumin in PBS). Cells were washed three times with 0.05% Tween-20 in PBS and stored in PBS until examination on a fully automated Zeiss inverted microscope (AxioObserver Z1) equipped with a MS-2000 stage (Applied Scientific Instrumentation, Eugene, Orlando, USA), a CSU-X1 spinning disk confocal head (Yokogawa) and a LaserStack Launch with selectable laser lines (Intelligent Imaging Innovations, Denver, CO). Images were acquired using a CoolSnap HQ camera (Roper Scientific, Planegg, Germany), a 63x oil objective (Plan Neofluoar 63x/1.25) and the Slidebook software (version 6.0; Intelligent Imaging Innovations, Denver, CO). Images were processed with Adobe Photoshop CS4 (Adobe Systems, Mountain View, California, USA).

### CD47 expression in AML patients

CD47 expression levels on primary AML samples were analyzed by flow cytometry (Navios, Beckman Coulter, Krefeld, Germany) using the mAbs CD47 (clone 472603, R&D), CD45 (clone J33, Beckman Coulter), CD34 (clone 581, Beckman Coulter) and CD38 (clone LS198-4-3, Beckman Coulter) and the corresponding isotype controls. Bulk AML cells were identified by limiting the analysis of primary AML sample material to SS^Clow^/CD45^dim^ cells. LSCs were defined as a CD34^+^/CD38^−^ subgroup of SS^Clow^/CD45^dim^ cells. As a measure for CD47 intensity, CD47 MFI ratio was calculated using FlowJo software (Version 9.6, Tree Star Inc., Ashland, Oregon).

### Antibody-dependent cellular cytotoxicity (ADCC) and preferential killing assays

Target cells (MOLM-13 or SEM) were labeled with calcein AM (Thermo Fisher Scientific) according to manufacturer's protocol. Calcein-labeled target cells were incubated with freshly isolated or IL-2 expanded NK cells in an effector-to-target (E:T) ratio of 2:1 and licMABs or mAb at different concentrations for 4 h. Target cells were cultured in 10% Triton X-100 to assess the maximum unspecific lysis. Calcein release was measured by fluorescence intensity with an Infinite^®^ M100 plate reader instrument (TECAN, Männedorf, Switzerland) and specific lysis was calculated as follows:

$$\text{Specific lysis }(\%)\;=\frac{{\text{Fluorescence}}_{\text{Sample}}{\text{ -Fluorescence}}_{\text{Spontaneous lysis}}}{{\text{Fluorescence}}_{\text{Maximum lysis}}{\text{-Fluorescence}}_{\text{Background}}}\times 100$$

Averaged specific lysis of triplicates or quadruplicates were plotted according to a dose-response curve and analyzed using the integrated four parameter non-linear fit model.

To assess the preferential killing of licMABs, an ADCC assay was performed with a 1:1 mixture of MOLM-13 and OCI-AML3 cells as target cells. Two assays, one containing Calcein-labeled MOLM-13 cells and unstained OCI-AML3 cells and the other unstained MOLM-13 cells and Calcein-labeled OCI-AML3 cells were executed in parallel using the same freshly isolated NK cells. Preferential killing was evaluated using licMABs and antiCD33 mAb at concentrations of 10 μM or the previously described EC_50_ and specific lysis was measured and calculated as mentioned above. This experiment was repeated three times using three different donors and mean and SEM values were plotted.

### Antibody-dependent cellular phagocytosis (ADCP)

Phagocytosis assay was performed as described previously [53]. Briefly, isolated monocytes were stained with PKH67 (Sigma-Aldrich) according to the manufacturer's instructions and differentiated to macrophages by 20 ng/ml Macrophage-Colony Stimulator Factor (M-CSF) (R&D Systems) in X-VIVO 10 medium (Lonza) supplemented with 10% autologous serum. MOLM-13 cells were stained with PKH26 (Sigma-Aldrich) following the manufacturer's instructions and incubated in a 1:2 E:T ratio with licMABs or mAb concentrations ranging from 0.01 nM to 100 nM for 2 h. Polybead^®^ Carboxylate Red-Dyed Microspheres of 6 μm (Polysciences) were used as a positive control and incubation either at 4°C or at 37°C in the presence of 10 μM Cytochlasin D (Sigma-Aldrich) served as a negative control. Cells were harvested, measured by imaging flow cytometry using an ImageStream^®^X Mark II instrument (Merck Millipore, Billerica, Massachusetts, USA) and analyzed with IDEAS^®^ and INSPIRE^®^ Software (Merck Millipore, Billerica, Massachusetts, USA). The maximum phagocytosis value was set to 100% and all data points were normalized accordingly. Mean values and standard errors of triplicates were calculated and plotted.

### Antibody-dependent cellular cytotoxicity in primary AML patient samples

*Ex vivo* expanded primary AML cells of 9 different patients were co-cultured with freshly isolated healthy donor NK cells, at an E:T ratio of 5:1 in an *ex vivo* long term culture system as described by Krupka and coworkers [29, 54]. Antibodies were added at a final concentration of 10 nM. After 24 hours, cells were harvested, stained for CD16 (clone B73.1), CD56 (clone HCD 56), CD33 (clone WM53) and in concrete cases CD123 (clone 6H6; all antibodies from Biolegend) and analyzed by flow cytometry with a BD LSR II (Becton Dickinson, Heidelberg, Germany). The percentage of residual CD33 or CD123 positive cells in treated cultures relative to control cultures was used to determine licMAB-mediated cellular cytotoxicity. Patient characteristics are summarized in Supplementary Table S1.

### Plotting and statistical analysis

Unless stated otherwise, data was analyzed and plotted with GraphPad Prism version 6.00 for Windows (GraphPad Software, La Jolla, California, USA).

Differences in CD47 expression on AML samples were calculated using the Mann-Whitney U test. Statistical differences in phagocytosis were assessed by an unpaired, parametric Student's *t-test* with Welch correction and statistical differences of patient responses were assessed by the Wilcoxon test. Statistical significance was considered for *p-value* < 0.05 (*), < 0.01 (**), < 0.001 (***) and < 0.0001 (****).

## SUPPLEMENTARY MATERIALS FIGURES AND TABLES


