# Supplementary material for: SIRPα-antibody fusion proteins stimulate phagocytosis and promote elimination of acute myeloid leukemia cells
Source: Oncotarget. 2017 Jan 4;8(7):11284–301. doi: 10.18632/oncotarget.14500 (PMC5355265; doi:10.18632/oncotarget.14500)
Supplement: Supplementary file 1 [file oncotarget-08-11284-s001.pdf]

SIRPα-antibody fusion proteins stimulate phagocytosis and promote elimination of acute myeloid leukemia cells

Supplementary Materials

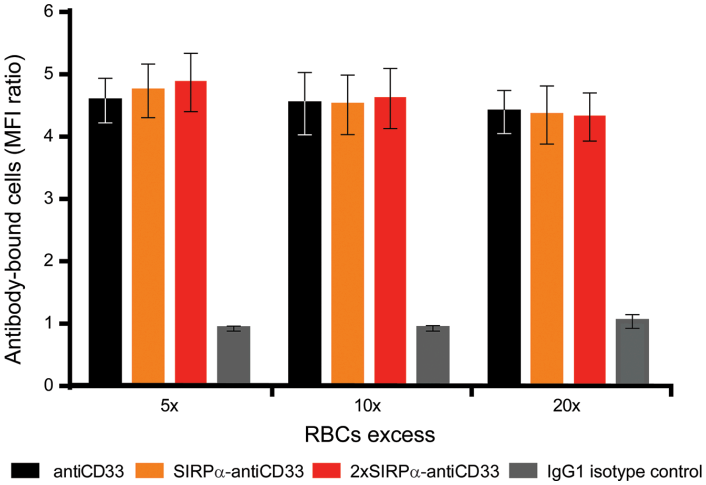

Supplementary Figure S1: LicMAB targeting is dictated by the high binding to CD33. MFI ratio of antibody-bound cells of the MOLM-13 and RBCs mixture was measured for antiCD33 mAb, SIRPα-antiCD33 licMAB, 2xSIRPα-antiCD33 licMAB and human IgG1 isotype control (sample / secondary staining). Error bars indicate SEM of three independent experiments with three different donors.

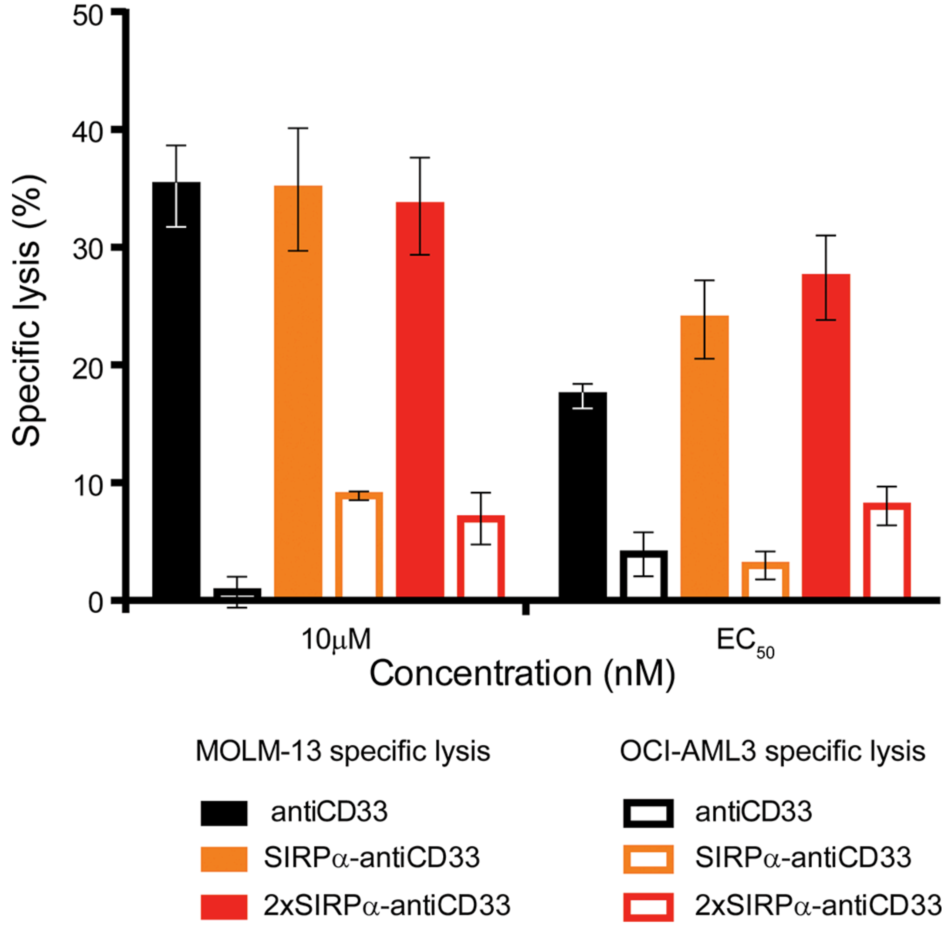

Supplementary Figure S2: LicMABs preferentially target and induce clearance of cells expressing high levels of CD33. Preferential lysis of MOLM-13 cells respect to OCI-AML3 cells is mediated by antiCD33 mAb, SIRPα-antiCD33 licMAB and 2xSIRPα-antiCD33 licMAB at concentrations of 10 nM and at the EC_{50}. Error bars indicate SEM of triplicates with three different donors.

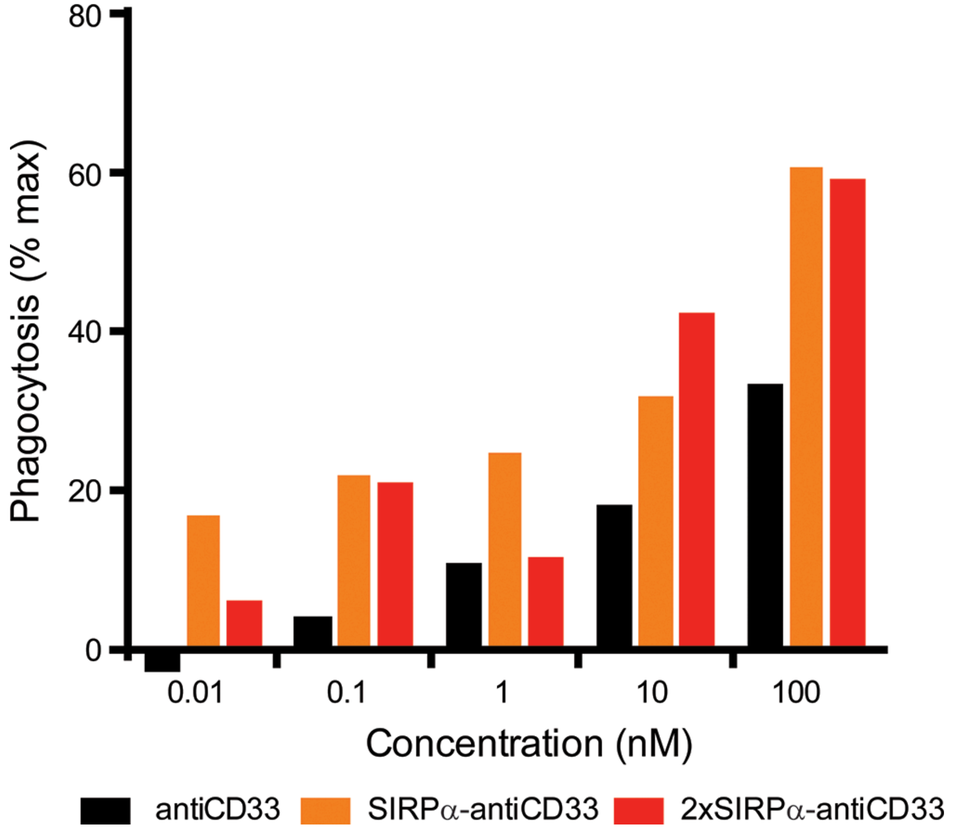

Supplementary Figure S3: LicMAB molecules stimulate phagocytosis of OCI-AML3 cells. antiCD33 mAb, SIRPα-antiCD33 licMAB and 2xSIRPα-antiCD33 licMAB induced phagocytosis of CD33 and CD47 positive AML-derived OCI-AML3 cell line by donor-derived human macrophages.

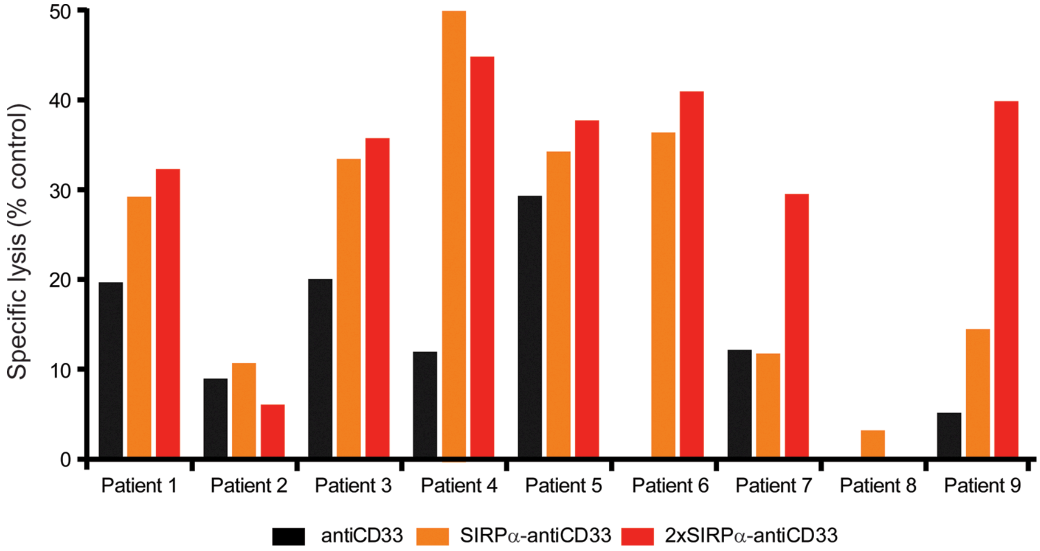

Supplementary Figure S4: Cytotoxicity of primary AML patient cells is mediated by licMABs. Individual responses to licMAB-induced NK cell-dependent cytotoxicity (summarized in Figure 7) of the 9 analyzed AML patient samples are depicted.

Supplementary Table S1: AML patient characteristics

PT	Gender	Disease Phase	Material	Blasts (%)	*NPM1* mut.	*FLT3-ITD*	Karyotype	ELN genetic group	CD33 MFI ratio	CD47 MFI ratio
**1**	F	ID	BM	91	+	–	normal	favorable	111.8	47.1
**2**	F	ID	BM	88	+	+	normal	intermediate I	110.0	76.3
**3**	M	ID	PB	89	–	+	47, XY, + 8	intermediate II	73.0	95.2
**4**	M	Relapse	BM	93	–	–	complex	adverse	52.3	34.5
**5**	M	ID	BM	71	–	–	normal	intermediate I	54.2	58.2
**6**	M	ID	BM	94	–	–	normal	intermediate I	12.6	75.9
**7**	W	Relapse	BM	91	–	+	46,XX; t (5,11)	Intermediate II	90.0	27.1
**8**	M	ID	PB	71	–	–	46,XX; der (16) t (1;16) (q12;q21)	Intermediate II	37.0	31.7
**9**	W	ID	BM	n.a.	+	+	n.a.	n.a.	n.a.	n.a.

PT, patient; M, male; F, female; ID, initial diagnosis; PB, peripheral blood; BM, bone marrow; mut, mutation; ELN, European Leukemia Net; n.a., not available.
